# Characterisation of *Lactobacillus plantarum* of Dairy-Product Origin for Probiotic Chèvre Cheese Production

**DOI:** 10.3390/foods11070934

**Published:** 2022-03-24

**Authors:** Achirawit Ngamsomchat, Thida Kaewkod, Maytiya Konkit, Yingmanee Tragoolpua, Sakunnee Bovonsombut, Thararat Chitov

**Affiliations:** 1 Division of Microbiology, Department of Biology, Faculty of Science, Chiang Mai University, Chiang Mai 50200, Thailand; achirawit_ng@cmu.ac.th (A.N.); tda007suju@gmail.com (T.K.); yingmanee.t@cmu.ac.th (Y.T.); sakunnee.b@cmu.ac.th (S.B.); 2 Division of Microbiology, Faculty of Science and Technology, Nakhon-Pathom Rajabhat University, Nakhon-Pathom 73000, Thailand; maytiyakonkit@webmail.npru.ac.th; 3 Environmental Science Research Center (ESRC), Chiang Mai University, Chiang Mai 50200, Thailand

**Keywords:** probiotic, functional food, lactic acid bacteria, dairy, fermented food, goat cheese, fresh cheese

## Abstract

Probiotics are increasingly used as functional food ingredients. The objectives of this study were to isolate and characterise probiotic bacteria from dairy and fermented foods and to use a selected strain for the production of probiotic chèvre cheese. Tolerance to acid (pH 2.0) and bile salt (0.4% (*w*/*v*)) were first investigated, and then other probiotic properties were determined. Out of 241 isolates, 35 showed high tolerance to acid and bile salt, and 6 were chosen for further characterisation. They were *Lactobacillus plantarum* and *L. fermentum*, and possessed antibacterial activities against foodborne pathogens such as *Bacillus* *cereus*, *Staphylococcus* *aureus*, *Salmonella* *enterica* and *Escherichia* *coli* O157:H7. *L. plantarum* (isolate AD73) showed the highest percentage of adhesion (81.74 ± 0.16%) and was nontoxic to Caco-2 cells at a concentration of 10^8^ CFU/mL. This isolate was therefore selected for the production of probiotic chèvre cheese from goat’s milk and was prepared in a lyophilised form with a concentration of probiotic culture of 8.6 log CFU/g. The cheese had a shelf life of 8 days. On the expiry date, the probiotic, the starter and the yeast contents were 7.56 ± 0.05, 7.81 ± 0.03 and 5.64 log CFU/g, respectively. The level of the probiotics in this chèvre cheese was still sufficiently high to warrant its being a probiotic cheese.

## 1. Introduction

Probiotic foods have been in high and increasing demand in recent years. The global value of probiotic foods was USD 14.9 billion in 2007, USD 16 billion in 2008 and USD 19.6 billion in 2013. The global value of probiotic food products is estimated to increase by 7% annually, and by 2023 it is estimated to reach USD 69.3 billion [1,2]. Probiotic bacteria are one of the most important ingredients in functional food products [3]. Probiotics are defined as living organisms which, when taken in an appropriate amount into the body, produce health benefits [4]. They have the potential to control and reduce metabolic disorders in the body. They can also reduce disorders and the risk of developing diseases, such as gut disorders [5], diseases caused by urogenital infections [5], acute diarrhoea [6] and lactose intolerance [7]. They can also decrease postoperative complications [8], and many are known to have antimicrobial activities [9], anti-colorectal cancer activities [10], and to prevent gastrointestinal disorders [11]. Moreover, probiotics are an important source of antioxidants, helping to reduce many chronic diseases caused by oxidative reactions. When the immune system is impaired due to various pathogenic microorganisms, increasing the intake of probiotics instead of antibiotics is a good solution. Thus, probiotics are cost-effective functional food ingredients and are highly beneficial in improving human health [12]. 

A probiotic strain must be tolerant to various stresses, such as low pH and a high concentration of bile in the intestine [13]. Different bacterial species have different degrees of tolerance. For example, the genus *Lactobacillus* is more tolerant to low pH conditions and has a higher survival rate than *Bifidobacterium* [6]. Many probiotic strains can produce inhibitory substances against pathogenic microorganisms, which include organic acids, hydrogen peroxide (H_2_O_2_), bioactive peptides (bacteriocins), and diacetyl through the metabolism of biomolecules such as carbohydrates, proteins, and other compounds. Other antimicrobial mechanisms have also been found, including competition, co-aggregating, and immunity stimulation [14]. The adhesion of probiotics to human epithelial cells brings about microbial colonisation. The adhesion of microbial cells to human epithelial cells depends on the molecules on the surface of the probiotic species. In addition, the adhesion factor depends on the electrostatic balance, Van der Waals relations on object surfaces, and bacterial extracellular components [15,16]. The adhesion assays of possible probiotics can be tested using mammalian epithelial cells, including Caco-2 cells, HT-29 cells, and fetal I-407 cells [6]. 

Although many probiotics had been isolated from healthy humans, new sources of novel probiotic species are being explored [17]. International food safety organisations and those from the United States and Canada (WHO, US FDA and Health Canada) have established principles for assessment of the safety of human probiotics [5]. Many probiotic foods are manufactured in the dairy industry, such as yoghurt, cheeses and kefir. These foods are recognised for their benefits to the host’s body functions [18,19]. Bacteria that are commonly used as probiotics in commercial products include members of the genera *Bifidobacterium* and *Lactobacillus*. Species that have been noted for their probiotic properties among lactobacilli include *Lactobacillus acidophilus*, *L. casei*, *L. crispatus*, *L. fermentum*, *L. gasseri*, *L. johnsonii*, *L. paracasei*, *L. plantarum*, *L. reuteri*, *L. rhamnosus* and *L. helveticus*. Members of *Lactococcus lactis* and members of bifidobacteria, including *Bifidobacterium bifidum*, *B. breve*, *B. infantis*, *B. longum*, *B. lactis*, *B. adolescentis*, *B. essensis* and *B. laterosporus*, have also been recognised as probiotics. Moreover, other species that could be used as probiotic bacteria are *Escherichia coli Nissle*, *Saccharomyces boulardii*, *Streptococcus thermophilus*, *Enterococcus francium*, *Propionibacterium*, *Pediococcus* and *Leuconostoc* [20,21,22]. 

In this study, we aimed to isolate probiotic bacteria from potential food sources, especially dairy and fermented foods, to investigate their probiotic properties and to demonstrate the incorporation of a high-potential probiotic isolate into a goat cheese product.

## 2. Materials and Methods

### 2.1. Isolation of Potential Probiotic Bacteria from Dairy and Fermented Foods 

Eight types of samples of dairy and fermented foods were collected for the isolation of potential probiotic bacteria. The samples and their sources are shown in Table 1. A portion (25 g) of each sample was homogenised in 225 mL of 0.1% peptone water using a stomacher. The samples were serially diluted using the same diluent to achieve the dilutions of 10^−1^–10^−8^. From the serially diluted samples, probiotic bacteria were isolated by spread plating on MRS-cysteine-bromophenol blue (MRS-Cys-BPB) agar. The plates were incubated at 37 °C for 48 h under an anaerobic condition (created by placing a gas pack in an anaerobic jar). After that, the colonies of the microbial isolates were re-streaked on MRS-Cys-BPB agar to obtain pure cultures, which were stored on MRS-Cys agar slant at 4 °C or as frozen cultures.

### 2.2. Investigation of Probiotic Properties of Isolates

**Figure 1:**
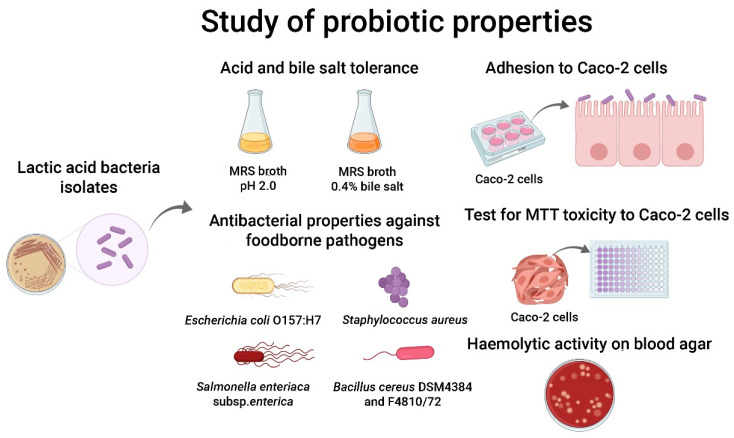
Investigation of the probiotic properties of dairy and fermented food isolates.

The isolates obtained from various food samples were investigated for their probiotic properties to select potential and suitable isolates that would be used in the production of a functional food product. A diagram summarising the tests for probiotic properties is shown in Figure 1.

#### 2.2.1. Acid Tolerance

The acid tolerance of the bacterial cultures was investigated in MRS broth (pH 2.0, adjusted using 1M HCl, which resembles the stomach pH). Each of the isolates were subcultured into MRS broth and incubated overnight at 37 °C under an anaerobic condition. The cultures were then transferred (10% (*v*/*v*)) into fresh MRS broth and incubated at 37 °C for 3 h, according to the possible transit time through the stomach. The number of the bacteria that survived was measured using the spread-plating method on MRS-Cys-BPB agar and compared with the initial numbers of the isolates before incubation. The tolerance percentage is calculated as follows (Equation (1)) [11]:

(1) $$\mathrm{Tolerance}\;\mathrm{percentage}\;=\frac{\mathrm{number}\;\mathrm{of}\;\mathrm{suvivors}}{\mathrm{initial}\;\mathrm{number}\;\mathrm{of}\;\mathrm{bacteria}}\times 100$$

#### 2.2.2. Bile Salt Tolerance 

The bile salt tolerance of the cultures was investigated in MRS broth supplemented with 0.4% (*w*/*v*) ox bile. The cultures were inoculated as above (2.2.1) and incubated at 37 °C for 3 h under an anaerobic condition. The tolerance to bile salt was measured and the tolerance percentage calculated as above.

#### 2.2.3. Haemolytic Activity Test 

The isolates were tested for haemolytic activity on Columbia blood agar plates supplemented with 5% sheep blood. After 48 h of incubation at 30 °C, the plates were examined for haemolytic reactions [23]. 

#### 2.2.4. Adherence to Caco-2 Cells

Potential adhesion of the bacterial isolates to intestinal cells was investigated using a human colorectal carcinoma (Caco-2) epithelial cell model. The cell line was grown in Dulbecco’s Modified Eagle Medium (DMEM) supplemented with 10% heat-inactivated fetal bovine serum, penicillin (100 units/mL) and streptomycin (100 µg/mL). Caco-2 cells were adjusted 1 × 10^5^ cells per well and cultured in 6-well microplates for 24 h at 37 °C in a 5% CO_2_ incubator. The overnight cultures of the bacterial isolates were harvested by centrifugation, washed twice with phosphate buffer saline (PBS, pH 7.4), and resuspended in the same buffer to have a turbidity equal to that of the McFarland standard no. 0.5, which had a concentration of approximately 10^8^ CFU/mL. Then, the bacterial cells were added into each well containing the Caco-2 cells, and the plates were incubated for 3 h. After incubation, nonadherent bacterial cells were removed, and the cells were subsequently washed three times with PBS. The adherent bacteria on the Caco-2 cells were fixed with methanol for 5 min and stained with Giemsa for 15 min before observation under the microscope. Moreover, the adherent bacteria were detached using 0.05% Trypsin-EDTA, serially diluted, and spread on MRS-Cys-BPB agar. The plates were incubated at 37 °C for 48 h. The percentage of bacterial adhesion is calculated as follows (Equation (2)) [24]:

(2) $$\mathrm{Adhesion}\;\mathrm{percentage}\;=\frac{\mathrm{number}\;\mathrm{of}\;\mathrm{adhered}\;\mathrm{bacteria}\;}{\mathrm{initial}\;\mathrm{number}\;\mathrm{of}\;\mathrm{bacteria}\;\mathrm{added}}\times 100$$

#### 2.2.5. Antibacterial Activity Test

Using the agar well diffusion method, the antibacterial activity of acid- and bile salt-tolerant isolates against some foodborne pathogens was determined [25]. The foodborne pathogens included *Escherichia coli* O157:H7, *Salmonella enterica* subsp. *enterica*, *Staphylococcus aureus*, *Bacillus cereus* DSM4384 (a diarrhoeagenic strain) and F4810/72 (an emetic strain). Antibacterial activity was tested using the cell suspension and cell-free supernatant from the overnight cultures of the isolates (the latter was prepared by centrifugation of the cell suspension at 10,000 rpm for 10 min at 4 °C and the supernatant was filtered through a 0.22-µm membrane filter). Penicillin (100 IU for *B. cereus* and 1 IU for *S. aureus*) and polymyxin B (50 µg/mL) were used as positive controls. 

#### 2.2.6. Test for Toxicity of Bacterial Isolates to Caco-2 Cells 

The Caco-2 cells were cultured in a 96-well plate and incubated for 24 h at 37 °C with 5% CO_2_. After that, a 100 µL portion of the cell suspension of each bacterial isolate was added to each well. The microplate was then incubated for 24 h at 37 °C with 5% CO_2_. Then, the microplate was washed with 1× phosphate buffer saline (PBS, pH 7.2) and 1 mL of DMEM supplemented with 2 mg/mL gentamycin was added to each well. The microplate was then incubated for 24 h with 5% CO_2_. The solution was then removed and MTT (30 µL of 2 mg/mL) was added to each well, and the microplate was further incubated for 4 h. DMSO (100 µL) was added to dissolve the crystalline formazan precipitates. The absorbances at 540 nm and 630 nm were measured in triplicate using a microplate reader. The percentage of survival was calculated (Equation (3)) and compared to the control cells (without bacteria) [26,27].

(3) $$\mathrm{Percentage}\;\mathrm{of}\;\mathrm{cell}\;\mathrm{survival}\;=\frac{\;\mathrm{OD}\;\mathrm{at}\;570\;\mathrm{nm}-\mathrm{OD}\;\mathrm{at}\;630\;\mathrm{nm}\;\mathrm{of}\;\mathrm{treated}\;\mathrm{cells}}{\;\mathrm{OD}\;\mathrm{at}\;570\;\mathrm{nm}-\mathrm{OD}\;\mathrm{at}\;630\;\mathrm{nm}\;\mathrm{of}\;\mathrm{control}\;\mathrm{cells}}\times 100$$

### 2.3. Characterisation and Identification of Bacterial Isolates

The isolates that possessed probiotic properties (from the tests above) were subjected to Gram staining and biochemical assays, including catalase test and carbohydrate fermentation test (using API 50 CHL, bioMérieux, Marcy l’Etoile, France). The potential probiotic isolates were identified using *16S rRNA* gene sequencing. To do this, DNA was extracted from the overnight cultures using the DNeasy Blood and Tissue kit (Invitrogen, CA, USA) following the manufacturer’s instructions. PCR amplification of the *16S rRNA* gene was performed using primers 27F (5′-AGAGTTTGATCMTGGCTCAG-3′) and 1492R (5′-CGGTTACCTTGTTACGACTT-3′) [24]. The reaction conditions were as follows: initial denaturation at 94 °C for 2 min; 35 cycles of denaturation at 94 °C for 30 s, primer annealing at 55 °C for 30 s and extension at 72 °C for 30 s; and a final extension at 72 °C for 7 min (Lane, 1991) [28]. The amplified gene fragments were purified using a purification kit (Vivantis, Shah Alam, Selangor Darul Ehsan, Malaysia) and subjected to DNA sequencing (Celemics, Seoul, Korea). The sequences were aligned with the nucleotide sequences in the NCBI database using the BLAST algorithm. The isolates were identified according to the percentage identity with the reference sequences. 

### 2.4. Preparation of Probiotic Culture

The selected probiotic isolate was cultured in 100 mL of MRS-Cys broth for 48 h, then centrifuged at 10,000 rpm for 15 min at 4 °C. The cell pellet was washed twice using phosphate buffer saline (PBS, pH 7.2) and resuspended in 100 mL of 5% (*w*/*v*) skim milk. The cell suspension was freeze-dried in a lyophiliser (Labconco, MO, USA) [29]. A plate count was performed to determine the quantity of the culture. This lyophilised culture, having a concentration of 8.6 log CFU/g, was used in manufacturing probiotic chèvre cheese.

### 2.5. Production of Probiotic Chèvre Cheese

A potential probiotic culture (*Lactobacillus plantarum* AD73), selected based on its identity and probiotic properties, was used to develop a probiotic chèvre cheese, which, in this study, was a fresh goat’s milk cheese. The milk used to manufacture the cheese was bulk goat’s milk from one farm, which was a mixture of morning milk and evening milk collected the day before. The milk was put in an ice box immediately after milking and was also transported in an ice box, so it was kept cool at all times until the time of the manufacturing of the cheese (within 36 h of the first milking time for this batch). 

**Figure 2:**
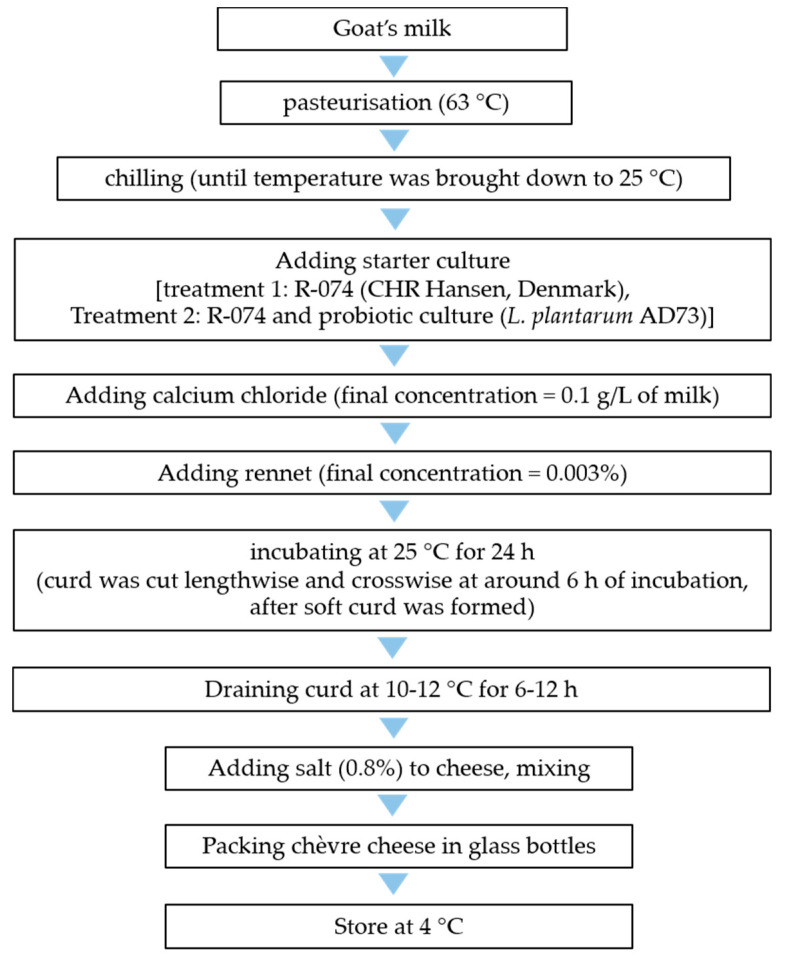
Production of chèvre cheese with and without probiotic culture.

The chèvre cheese was produced with 2 treatments: non-probiotic and probiotic. Treatment 1, the non-probiotic treatment or the control, was produced using a mesophilic cheese starter culture (R-704 supplied by CHR Hansen, Denmark) containing a mixed culture of *Lactococcus lactis* subsp. *lactis* and *Lactococcus lactis* subsp. *cremoris*). Treatment 2, or the treatment with probiotic culture, was produced using the R-704 starter with the addition of *L. plantarum* AD73, prepared as above (2.4). Each treatment of chèvre cheese was produced from 12 kg of goat’s milk. The raw milk was briefly pasteurised at 63 °C. When the temperature reached this point, it was immediately cooled with iced water. The starter culture was used at the proportion recommended by the manufacturer, which was 1 U per 10 litres. CaCl_2_ (dissolved in water before use) was added after the starter and probiotic culture at a concentration of 0.1 g/l. Rennet (powder form, dissolved in water before use) was added to the milk at a concentration of 0.003%. For treatment 2, the lyophilised culture AD73 and the mesophilic cheese starter culture were added at the same concentration as for treatment 1 with a ratio of 1:1. The milk mixture was incubated at 25 °C for approximately 6 h, until a soft curd was formed. The curd was then cut lengthwise and crosswise and was further incubated at 10 ± 2 °C for another 18 h. After that, the curd was transferred to a cheesecloth and allowed to drain for approximately 12 h, before being salted. The manufacturing process of chèvre cheese is summarised in Figure 2 (a method modified from that of Carunchiawhetstine [30]).

### 2.6. Moisture Content and pH Analysis

To prepare a cheese sample for pH measurement, the sample was mixed with distilled water at a ratio of 1:1 and homogenised using a sample homogeniser (Stomacher). The pH of chèvre cheese was monitored for 14 days using a pH meter. The moisture contents of the chèvre cheese made with and without the probiotic culture were analysed using a moisture balance (Precisa, Dietikon, Switzerland). Each of the triplicate spectra was measured at a different zone of the flat surface in the spectral region of 1100 to 2500 nm with 1 nm resolution. The instrument operated in a diffuse reflectance mode, with its signals expressed as percentages [31]. The measurements were taken in three replicates.

### 2.7. Microbiological Analysis of Chèvre Cheese

The two treatments of Chèvre cheese (without and with the probiotic culture, or treatments 1 and 2, respectively) were subjected to microbiological analyses, including total viable count, total lactic acid bacteria, yeast and mould count and probiotic count (*L. plantarum* AD73, performed for treatment 2 only). The cheese samples (25 g of each) were homogenised in 225 mL of 0.1% peptone water and then serially diluted to a dilution of 10^−8^. The total viable count was performed with the diluted samples using the drop plate method on Plate Count agar and incubated at 30 °C for 48 h. The numbers of lactic acid bacteria and probiotic bacteria were investigated using the spread-plate method on MRS-Cys-BPB agar. The plates were incubated under an anaerobic condition at 37 °C for 48 h. Yeast and mould counts were performed using the spread plate method on DRBC agar and incubated at 25 °C for 5 days. The counts were expressed as the log number of probiotic bacteria per gram of the product (log CFU/g). All microbiological analyses were performed in triplicate every 2 days for a total of 14 days. 

### 2.8. Statistical Analysis

The data were expressed as mean ± standard error of mean. Tukey’s HSD test with One-Way ANOVA was used to analyse the dependent and independent data. All statistical analyses were conducted using IBM SPSS statistics version 20.

## 3. Results

### 3.1. Isolation of Potential Probiotic Bacteria from Dairy and Fermented Foods

The samples from which potential probiotic bacteria were recovered included pla-som (fermented fish), kimchi, pickled cabbage, tua-nao (alkaline-fermented soybean product), miang (fermented tea leaves), pickled garlic, kefir and raw goat’s milk. Representatives of bacterial colonies recovered from the MRS-Cys-BPB agar plates were selected for further analysis. 

A total of 241 bacterial isolates were recovered, and each one was tested for Gram stain reaction, cell morphology, catalase reaction and haemolytic activity. All the isolates were Gram-positive and catalase-negative (Table 2). They showed no haemolytic reaction on sheep blood agar (results not shown).

### 3.2. Acid and Bile Salt Tolerance 

All of the isolates recovered from the food samples were examined for their tolerance to acid and bile salt. Acid and bile salt tolerance tests were used to primarily screen for isolates that were potential probiotics. From the preliminary screening, it appeared that 35 out of 241 isolates had a high degree of tolerance (>50% survival) to acid and bile salt. 

**Figure 3:**
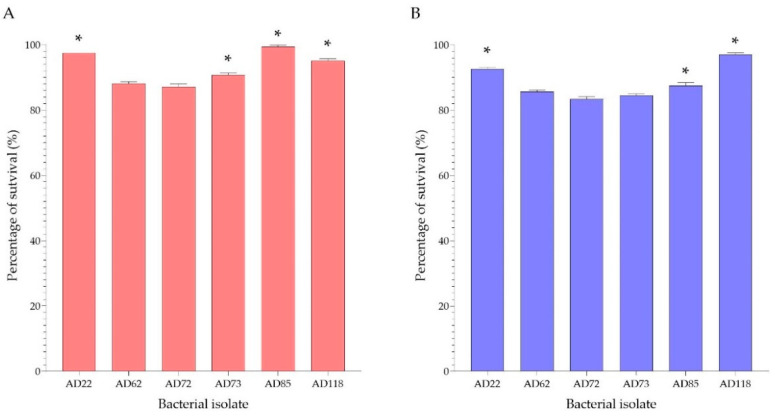
Tolerance to acid (pH 2.0) (**A**) and bile salt (0.4% (*w*/*v*)) (**B**) of six potential probiotic isolates, expressed as percentage of survival. * The percentage of survival was significantly different compared with the other isolates (*p* < 0.05; Tukey HSD test with One-Way ANOVA).

The 35 acid-bile salt tolerant isolates were Gram-positive, catalase- and oxidase-negative, with gamma haemolytic reaction (Table 2). These isolates were identified through their *16S rRNA* gene sequences as *Lactobacillus fermentum* and *Lactobacillus plantarum* (Table 3). Based on their acid/bile salt tolerance, only six isolates had more than 80% survival after being exposed to acid and bile salt (Figure 3). They were selected for further analysis for probiotic properties.

### 3.3. Carbohydrate Fermentation Profiles of Potential Probiotic Isolates

The selected potential probiotic bacterial isolates, including AD22, AD62, AD72, AD73, AD85 and AD118, were tested for carbohydrate fermentation using the API 50 CHL test kit. The carbohydrate fermentation profiles of the isolates are shown in Table 4. Both of the milk kefir isolates, AD72 and AD73, had a similar carbohydrate fermentation profile. It is interesting to observe that they could ferment more types of carbohydrates than the other isolates.

### 3.4. Antibacterial Activity Test

The selected isolates were tested for their antibacterial activity against foodborne pathogens using the agar well diffusion method, with a well diameter of 5 mm. The tests were carried out using cell suspension and cell-free supernatant of the isolates. The results showed that the cell suspension of the isolates had inhibition zones of 11–15 mm, and the cell-free supernatant of isolates had inhibition zones of 7–14 mm, as shown in Table 5 and Table 6.

From the results, it can be seen that the cell suspension and cell-free supernatant of all of the potential probiotic isolates had inhibitory effects against Gram-positive and Gram-negative pathogenic bacteria tested. In general, the cell suspension seemed to exhibit a higher inhibitory effect against the pathogens than the cell-free supernatant (Table 5 and Table 6). 

### 3.5. Adhesion of Bacterial Isolates on Caco-2 Epithelial Cells

**Figure 4:**
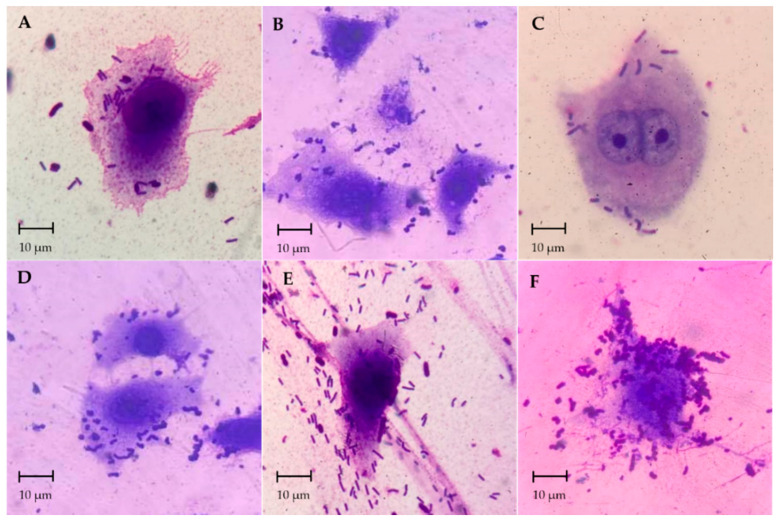
Adhesion of bacterial isolates on Caco-2 epithelial cells, (**A**); isolate AD22, (**B**); isolate AD62, (**C**); isolate AD72, (**D**); isolate AD73, (**E**); isolate AD85 and (**F**); isolate AD118. The cells were stained with Gimsa solution and observation under 100× objective lens.

**Figure 5:**
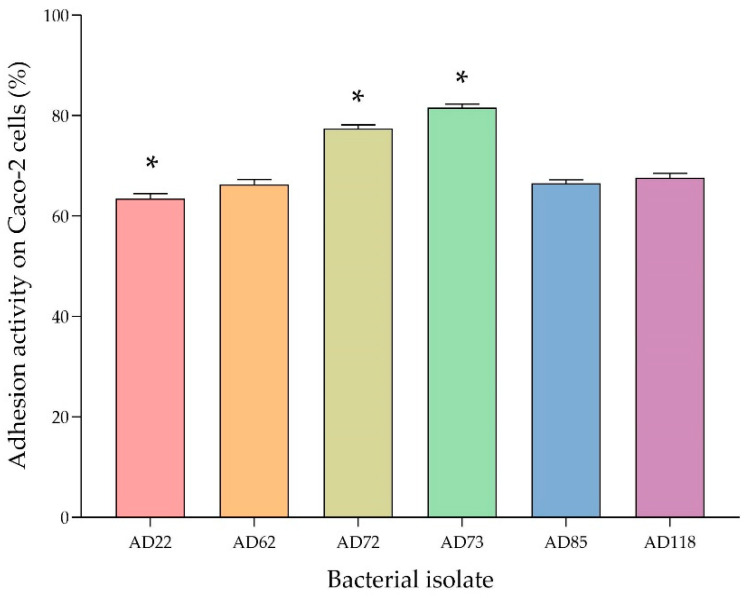
Adhesion activity of bacterial isolates on Caco-2 epithelial cells. The data presented in the figure are given as mean ± SD of triplicate experiments. * Statistically significant different compared with the other potential probiotic isolates (*p* < 0.05; Tukey’s HSD test with One-Way ANOVA).

The adhesion activity of probiotic isolates was examined on Caco-2 epithelial cells. All isolates could adhere to Caco-2 cells (Figure 4A–F). The AD73 isolate had the highest adhesion activity on Caco-2 cells, having a percentage of adhesion of 81.74 ± 1.6%, followed by the AD72 isolate, which had a percentage of adhesion of 77.06 ± 2.0%. The other isolates, including AD22, AD62, AD85 and AD118, were also shown to have more than 60% adhesion activity on Caco-2 cells (Figure 5).

### 3.6. Cytotoxicity to Caco-2 Cells

**Figure 6:**
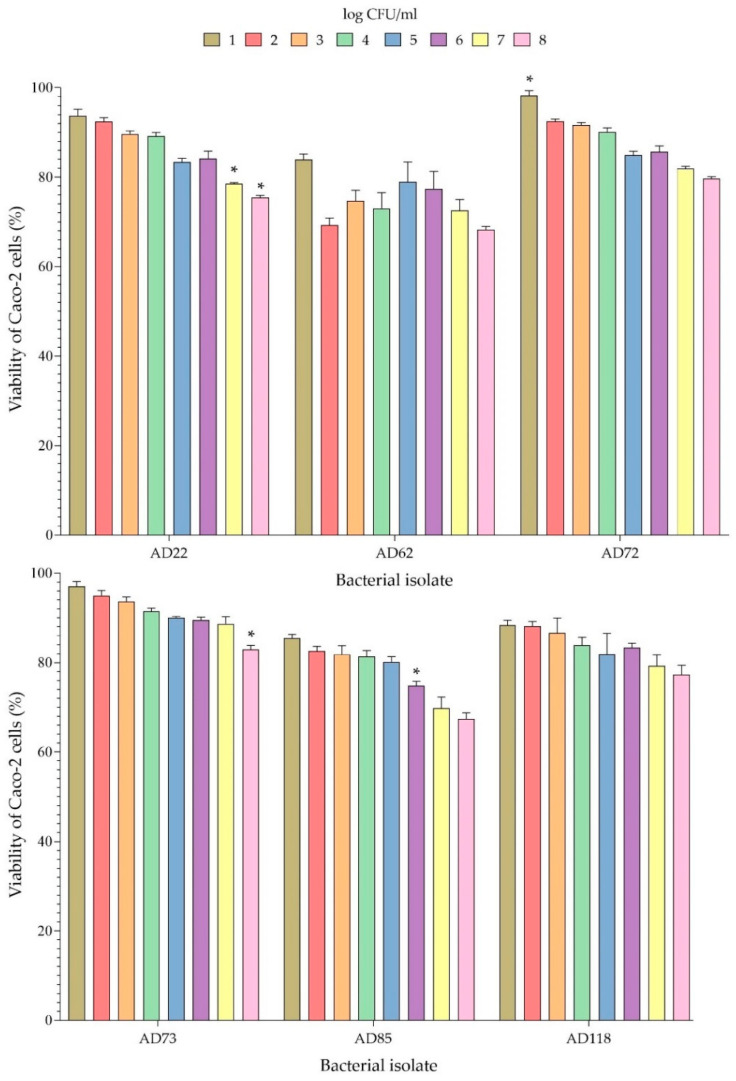
Survival of Caco-2 cells in the presence of probiotic bacterial isolates. * Statistically significant different compared with the other potential probiotic isolates (*p* < 0.05) (Tukey’s HSD test with One-Way ANOVA).

In order to evaluate the toxicity of the potential probiotic isolates on Caco-2 cells, the Caco-2 cells were incubated with 1–8 log CFU/mL of each bacterial isolate for 24 h. The viability of Caco-2 cells after being exposed to the probiotic bacterial isolates is presented in Figure 6. It can be seen that the AD73 isolate at a concentration of 8 log CFU/mL did not greatly affect the viability of Caco-2 cells, which still had a percentage of survival of 83.27%. Caco-2 cells exposed to AD72 at a concentration of 8 log CFU/mL also showed a high percentage of survival of 79.18%. Moreover, high survival of Caco-2 cells (more than 80%) was observed when tested with bacterial isolates AD22, AD62, AD85, AD118 with a number of bacterial cells of ≤6, ≤1, ≤5 and ≤6 log CFU/mL, respectively (Figure 6). Isolate AD62 seemed to be more toxic to Caco-2 cells than the other isolates.

### 3.7. Use of Selected Probiotic Culture for Production of Probiotic Chèvre Cheese

#### 3.7.1. Selection and Preparation of Probiotic Bacteria 

Various tests for probiotic properties showed that the AD73 isolate from milk kefir had the highest potential to be a probiotic. AD73 had the highest ability to adhere to Caco-2 cells, and it was also nontoxic to Caco-2 cells at a concentration of 8 log CFU/mL (the percentage of cell viability was 82.91%). These are considered important probiotic properties and are an indication that the isolate can be used as a probiotic in food products. In addition, it was identified as *Lactobacillus plantarum*, which is a well-known probiotic species that is on the approved probiotic list and is known to be suitable for use in dairy products. The selected probiotic isolate was prepared into a lyophilised culture, having a live probiotic number of 8.6 log CFU/g.

#### 3.7.2. Production of Chèvre Cheese

*L. plantarum* isolate AD73, was selected for use in the production of a novel probiotic chèvre cheese. The total bacterial count of the milk was 3.46 log CFU/mL. From 12 kg of goat’s milk, the volume of whey was 7.19 L for both treatments. The weight of the cheese was 2.185 kg (18.20%) and 2.010 kg (16.75%) for treatments 1 and 2, respectively. The moisture content of treatment 1 was slightly higher than treatment 2. The textures of the chèvre cheese of the two treatments were slightly different; the cheese of treatment 1 was more homogenous and had a smoother texture, whereas in treatment 2, lumps were found in the curd and the texture of the cheese was drier. The lumpy texture was less obvious after kneading the curd when salt was added (Table 7).

### 3.8. Microbiological Analysis of Chèvre Cheese

Microbiological analyses of the chèvre cheese products were performed to determine the survival of probiotics and the amounts of other microorganisms. Chèvre cheese is a soft cheese that does not require ripening and is intended for consumption as fresh cheese without prior heating. So, determination of the quantity and type of microorganisms is important, especially the number of bacteria involved in cheese production and spoilage microorganisms. In order to evaluate the survival of the probiotic culture and the shelf life of the chèvre cheese with and without probiotics, the pH and the microbial contents of the chèvre cheese were investigated every 2 days for a total of 14 days during storage at 4 °C. It was found that only treatment 1 had a pH decrease after storage (from 4.42 to 4.28) (Table 8). The microbial contents analysed included total viable count, number of lactic acid bacteria, probiotic bacteria, and the number of yeasts and moulds (evaluated as the main groups of spoilage microorganisms for this product). Moreover, the number of bacteria in both treatments changed during the period of cold storage. There were gradual decreases in the numbers of lactic acid bacteria and probiotics, while the number of yeasts gradually increased (Table 8 and Table 9). The alcoholic smell, which was detected on day 10 in both treatments, corresponded to the number of yeasts of approximately 6 log CFU/g.

## 4. Discussion

### 4.1. Isolation of Potential Probiotic Bacteria, Morphological, Biochemical and Identification of Isolates

Many types of dairy and fermented food samples, such as pla-som, pickled cabbage, pickled garlic, kimchi, tua-nao, miang, raw goat’s milk and milk kefir, appeared to be a potential food source of probiotic bacteria. Most of the bacterial isolates recovered from these foods were Gram-positive bacteria. They did not produce catalase and showed no haemolysis on sheep blood agar plate, which indicates that they might be lactic acid bacteria and are unlikely to be pathogenic. The nonpathogenic property is one of the most crucial criteria in the selection of a probiotic strain. 

The carbohydrate fermentation showed that all isolates could ferment L-Arabinose, D-Ribose, D-Glucose, D-Fructose, D-Mannose, Arbutin, D-Saccharose, and D-Raffinose. Isolates AD72 and AD73 from kefir could ferment more types of carbohydrates than the other potential probiotic isolates. They also had a similar carbohydrate fermentation profile, pointing to the possibility that they were the same strain or very closely related. The acid/bile salt-tolerant isolates were identified as *Lactobacillus plantarum* and *L. fermentum*. These species are considered suitable for use as probiotics due to their ability to survive in stressful environments and their nontoxic property [21,22].

### 4.2. Acid and Bile Salt Tolerance 

Out of 241 isolates, only 35 showed potential probiotic properties from the primary screenings, which included acid and bile salt tolerance. The survival of probiotic bacteria is essential for exerting health benefits on the host’s bodily functions. They must remain alive in the gastrointestinal (GI) tract to reach the large intestines [26]. Therefore, the key to the survival of probiotic bacteria is their resistance to unsuitable environmental conditions, such as low pH in the stomach and a high bile salt concentration in the intestine [32,33]. These unfavourable conditions can negatively affect the cells of beneficial bacteria by causing damage to the cell membrane through the disruption of the lipid bilayer [34]. In a previous study, the survival of potential probiotic bacteria, such as the members of the genera *Lactobacillus*, *Lactococcus* and *Enterococcus*, grown under an acidic condition (pH 2.5) and a condition with 0.3% bile salt was investigated. The results showed survival rates of 81–85% under the acidic condition and 65–98% under the condition containing bile salt [35]. In our study, potential probiotic properties of the lactic acid bacterial isolates were preliminarily assessed under a highly acidic (pH 2.0) condition and a high (0.4% (*w*/*v*)) bile salt concentration for 3 h, which simulate the conditions in the GI tract [36,37]. We identified potential probiotics that had a high tolerance of more than 65% and more than 70% to acid and bile, respectively. Moreover, the acid/bile salt-tolerant isolates were selected based on their species and their high ability to tolerate stress conditions. Six isolates that demonstrated acid tolerance of 87.17–98.44% and bile salt tolerance of 74.17–98.18% belonged to the approved probiotic species. 

### 4.3. Analysis of Properties of Probiotics of Bacterial Isolates with High Probiotic Potentials

One of the main properties of a probiotic strain is its ability to suppress the proliferation of harmful organisms in the gut. In our study, the potential probiotic isolates that were tolerant to acid and bile salt were further tested for their antibacterial activity against foodborne pathogens. The pathogens that were chosen for the test included *Bacillus cereus* DSM4384, *B. cereus* F4810/72, *Staphylococcus aureus*, *Salmonella enterica* subsp. *enterica* and *Escherichia coli* O157:H7. All six potential probiotic isolates possessed antibacterial activity against all of the test pathogen strains. In most cases, the potential probiotic isolates in the form of cell suspension showed stronger inhibitory effects against the pathogens than in the form of cell-free supernatant. This finding agreed with other studies which demonstrated that the antibacterial activities of the cell suspension of probiotic cultures were higher than those of the cell-free supernatant [38,39]. Many strains of probiotic bacteria can produce a wide range of inhibitory metabolites, such as organic acids, hydrogen peroxide and bacteriocins [34,37]. Lactic acid and hydrogen peroxide can inhibit many Gram-negative bacteria [39].

Lactic acid bacteria, especially those in the genera *Lactobacillus*, *Lactococcus* and *Pediococcus* can produce several bacteriocins, such as lactacin B from *Lactobacillus acidophilus* [40,41], plantaricin 423 from *Lactobacillus plantarum* [42], pediocin ST18 from *Pediococcus pentosaceus* and nisin Q from *Lactococcus lactis* [43]. Besides the antibacterial activity, the adhesion of the bacterial isolates to epithelial cells was investigated, using Caco-2 as a model. All of the six isolates tested showed high degrees of adhesion (64–81.74%). *L. plantarum* AD73 had the highest adhesion activity on Caco-2 cells, with a percentage of adhesion of 81.74 ± 0.16%. Many strains of *Lactobacillus* were found to have the ability to adhere to Caco-2 cells. Caggia et al. previously reported that *L. paracasei* could adhere to Caco-2 cells by more than 90% [44]. Moreover, *Lactobacillus rhamnosus* was found to have an adhesion activity of 60–80% [24]. 

The potential probiotic bacterial isolates were also investigated for their cytotoxicity to Caco-2 cells. Many isolates at the highest bacterial concentration (8 log CFU/mL) showed toxicity effects on Caco-2 cells. However, we found that *L. plantarum* AD73 at a concentration of 8 log cfu/mL was not toxic to Caco-2 cells (83.27 ± 1.85% survival). In a related study on the toxicity of *Lactobacillus plantarum* on Caco-2 cells, the organism was shown to be nontoxic to the cells, resulting in a percentage of cell survival of more than 90% [45]. When testing for toxicity, if the percentage of viability of a cell culture exceeds 80%, the bacterial strain is considered nontoxic, according to ISO 10993–5 (2009) [46].

### 4.4. Use of Probiotic Culture in Chèvre Cheese Production

One way of introducing a probiotic bacterial strain into the body is through the incorporation of the probiotic into food. In this study, chèvre cheese, a soft cheese made with goat’s milk, was chosen as a functional food to be formulated with the selected probiotic culture. Because of the advantages it had over the other strains, *L. plantarum* AD73 was selected for this purpose. The probiotic cheese was compared with the nonprobiotic one. The addition of the probiotic culture seemed to have an effect on the moisture content of the cheese (lower moisture content). The pH of the probiotic chèvre cheese remained stable during 14 days of storage, while the nonprobiotic cheese had a gradual decrease of pH by approximately 0.2, which can affect the taste. Due to its impact on sensory evaluation, texture is an important characteristic to be evaluated for cheeses [47]. The textures of the chèvre cheese in this study seemed to be affected by the addition of the probiotic culture, which caused the cheese to be drier and less homogenous because of the lumpiness in the curd (observed by eye and touching). This might be related to the moisture. However, the overall texture of the probiotic cheese was still acceptable.

### 4.5. Survival of Probiotic and Shelf-Life Evaluation of Chèvre Cheese

The shelf life was estimated based on the numbers of lactic acid bacteria, probiotics, yeasts and moulds, together with flavour and other sensory qualities. In terms of host health benefits, the probiotic population should exceed 10^6^–10^7^ CFU/g in the product before consumption [8]. High levels of live probiotics at 10^7^ CFU/g are recommended in probiotic foods to have beneficial effects [48]. Factors affecting the survival of probiotics include pH, fat content, and the concentration and type of protein and sugar. Therefore, product formulation can be adapted to improve the survival of probiotics [49]. From our results, the number of lactic acid bacteria and probiotics in the chèvre cheese gradually decreased during the 14-day storage. On the other hand, the number of yeasts gradually increased. The viable numbers of lactic acid bacteria and probiotics on day 8 still met the recommended levels, which were 8.00 ± 0.03 log CFU/g and 7.56 ± 0.05 log CFU/g, respectively. 

In soft cheese products, yeast contamination can cause off-flavours, softening, gas production, discolouration, and swollen packages. *Debaryomyces hansenii* (*Candida famata*) is a highly salt-tolerant organism frequently found in salted meat products, cheeses and brined vegetables. It can cause surface biofilms and off-flavours. Moreover, related species such as *Yarrowia lipolytica*, *Kluyveromyces marxianus*, *Pichia membranifaciens*, *Candida*, *Rhodotorula*, and *Cryptococcus* spp. are frequently microorganisms of concern. Subsequently, yeasts are regarded as an important component of the maturation microbiota in many cheeses. Some fermenting yeasts, such as *K. marxianus*, are required in blue cheese production, whereas their presence can cause a defect in other cheese varieties. On the other hand, the by-product of *D. hansenii* and *Y. lipolytica* may change the typical appearance and sensory characteristics of cheeses without reducing their overall quality. The differences between a beneficial and a spoilage outcome may be related to the growth of each strain [50,51]. If the appearance or flavour of a cheese is atypical and does not meet the consumer’s expectations or if it does not have the specific sensory characteristics of a cheese variety, the cheese is considered spoiled [52]. In this study, the off-flavour was observed as an alcoholic smell on day 10. This corresponded to yeast counts of approximately 6 log CFU/g or higher. This level of yeast could be used as a microbial indicator for the spoilage of this type of cheese. Therefore, the shelf life of the probiotic chèvre cheese was estimated to be 8 days. Within this period, the amount of live *L. plantarum* was still sufficient to warrant the characteristics of a probiotic food product. 

## 5. Conclusions

From this study, six bacterial isolates from fermented food and dairy products with a high tolerance to acid and bile salt were characterised for their probiotic potential. From the *16S rRNA* gene sequencing, they were identified as *Lactobacillus* species. *L. plantarum* AD73, an isolate from kefir, was chosen because of its highest percentage of adhesion to Caco-2 cells and its nontoxic property. Its antibacterial activities against foodborne pathogens and its ability to ferment a wide variety of carbohydrates support its probiotic potential. *L. plantarum* AD73 was used to produce a novel probiotic chèvre cheese. The shelf life of the cheese was determined to be 8 days, mainly by the development of an alcoholic smell that corresponded to the yeasts level that increased to approximately 6.0 log CFU/g. By day 8, the number of live *L. plantarum* in the cheese was still high (>7.0 log CFU/g), sufficient for it to be beneficial for human health and to be classified as a probiotic food product.

## Figures and Tables

**Table 1:** Samples used for bacterial isolation.

Sample Category	Sample	No. of Sample	Source
Milk and dairy	Milk kefir	1	Russia
	Raw goat’s milk	5	Chiang Mai, Thailand
Fermented food	Pla-som (fermented fish)	3	Pa-Yao, Thailand
	Tua-nao (alkaline fermented soybean)	4	Chiang Mai, Thailand
	Pickled garlic	2	Chiang Mai, Thailand
	Pickled cabbage	3	Chiang Mai, Thailand
	Miang (fermented tea leaf product)	4	Chiang Mai, Thailand
	kimchi	3	Chiang Mai, Thailand

**Table 2:** Bacterial isolates recovered from dairy and fermented food samples.

Source		No. of Isolate	Gram Stain Reaction and Cell Morphology	Catalase Production
Fermented foods	Kimchi	29	Gram-positive, rod-shaped	negative
	Pickled cabbage	29	Gram-positive, rod-shaped	negative
		13	Gram-positive, short rod-shaped	negative
	Miang	13	Gram-positive, rod-shaped	negative
		17	Gram-positive, short rod-shaped	negative
		7	Gram-positive, spherical-shaped	negative
	Tua-nao	8	Gram-positive, rod-shaped	negative
		12	Gram-positive, short rod-shaped	negative
		12	Gram-positive, spherical-shaped	negative
	Pickled garlic	5	Gram-positive, rod shaped	negative
		8	Gram-positive, spherical-shaped	negative
	Pla-som	17	Gram-positive, rod-shaped	negative
		25	Gram-positive, short rod-shaped	negative
Milk	Raw goat’s milk	38	Gram-positive, rod-shaped	negative
Dairy product	Milk kefir	8	Gram-positive, rod-shaped	negative
Total		241		

**Table 3:** The closest species/strain of selected isolates according to their *16S rRNA* gene sequences.

Isolate	Source	Closest Species/Strain	% Identity	Accession Number of Closet Relative	Accession Number *
AD22	Kimchi	*Lactobacillus plantarum* strain JCM 1149	100.00	NR_115605.1	OM807265
AD62	Tua-nao	*Lactobacillus fermentum* strain CIP 102980	99.80	NR_104927.1	OM807266
AD72	Milk kefir	*Lactobacillus plantarum* strain JCM 1149	100.00	NR_115605.1	OM807267
AD73	Milk kefir	*Lactobacillus plantarum* strain JCM 1149	99.93	NR_115605.1	OM807268
AD85	Miang	*Lactobacillus fermentum* strain CIP 102980	99.80	NR_104927.1	OM807269
AD118	Raw goat’s milk	*Lactobacillus fermentum* strain CIP 102980	99.93	NR_104927.1	OM807270

* Accession number of the sequences of the bacterial isolates in this study, deposited in the NCBI database.

**Table 4:** Carbohydrate fermentation profiles of potential probiotic isolates tested using API 50 CHL kit.

Test	AD22	AD62	AD72	AD73	AD85	AD118
Control	-	-	-	-	-	-
Glycerol	-	+	-	-	-	-
Erythritol	-	-	-	-	-	-
D-Arabinose	-	-	-	-	-	-
L-Arabinose	+	+	+	+	+	+
D-Ribose	+	+	+	+	+	+
D-Xylose	-	-	-	-	-	-
L-Xylose	-	-	-	-	-	-
D-Adonitol	-	-	-	-	-	-
Methyl β-D-glucopyranoside	-	-	-	-	-	-
D-Galactose	+	+	+	+	+	-
D-Glucose	+	+	+	+	+	+
D-Fructose	+	+	+	+	+	+
D-Mannose	+	+	+	+	+	+
L-Sorbose	-	-	-	-	-	-
L-Rhamnose	-	-	-	-	-	-
Dulcitol	-	-	-	-	-	-
Inositol	-	-	-	-	-	-
D-Mannitol	-	-	+	+	-	-
D-Sorbitol	-	-	-	-	-	-
Methyl α-D-mannopyranoside	-	-	+	+	-	-
Methyl α-D-glucoside	-	-	-	-	-	-
N-Acetyl glucosamine	-	-	+	+	-	-
Amygdalin	-	-	+	+	-	-
Arbutin	-	-	+	+	-	-
Esculin ferric citrate salicin	-	-	+	+	-	+
D-Cellobiose	-	-	+	+	-	-
D-Maltose	-	-	+	+	-	-
Arbutin	+	+	+	+	+	+
D-Melibiose	+	+	+	+	+	-
D-Melibiose	+	+	+	+	+	-
D-Saccharose	+	+	+	+	+	+
D-Trehalose	-	-	+	+	-	-
Inulin	-	-	-	-	-	-
D-Melezitose	-	-	+	+	-	-
D-Raffinose	+	+	+	+	+	+
Amidon	-	-	-	-	-	-
Glycogen	-	-	-	-	-	-
Xylitol	-	-	-	-	-	-
Gentobiose	-	-	+	+	-	-
D-Turanose	-	-	+	+	-	-
D-Lyxose	-	-	-	-	-	-
Tagatose	-	-	-	-	-	-
D-Focose	-	-	-	-	-	-
D-Lucose	-	-	-	-	-	-
L-Fucose	-	-	-	-	-	-
D-Arabitol	-	-	-	-	-	-
Potassium gluconate	-	-	+	+	-	+
Potassium 2-keto gluconate	-	-	-	-	-	-
Potassium 5-keto gluconate	-	-	-	-	-	-

Note: (+) fermented, (-) not-fermented.

**Table 5:** Effects of cell suspension of potential probiotic bacteria isolates on food pathogens.

Isolate	Inhibition Zone (mm)				
	*B. cereus* DSM4384	*B. cereus* F4810/72	*Staphylococcus * *aureus*	*Salmonella enterica* subsp. *enterica*	*E. coli * O157:H7
AD22	12.3 ± 0.07	12.0 ± 0.20 *	12.5 ± 0.07	12.9 ± 0.03 *	11.9 ± 0.05
AD62	12.2 ± 0.06	15.0 ± 0.10 *	12.2 ± 0.05 *	12.1 ± 0.03	12.3 ± 0.10 *
AD72	14.3 ± 0.09 *	14.0 ± 0.15 *	12.7 ± 0.02	11.0 ± 0.05	13.5 ± 0.05 *
AD73	13.0 ± 0.05 *	11.3 ± 0.10 *	13.5 ± 0.03 *	11.9 ± 0.04	12.0 ± 0.05
AD85	14.6 ± 0.07 *	14.6 ± 0.10 *	11.7 ± 0.02 *	12.4 ± 0.06 *	13.1 ± 0.06 *
AD118	11.8 ± 0.09 *	11.6 ± 0.04 *	12.5 ± 0.02	11.2 ± 0.05	12.0 ± 0.05
Penicillin	21.0 ± 0.05	20.0 ± 0.03	22.0 ± 0.05	ND	ND
Polymyxin B	ND	ND	ND	16.0 ± 0.07	15.0 ± 0.07

* Statistically significant different compared with the other potential probiotic isolates (*p* < 0.05; Tukey’s HSD test with One-Way ANOVA). The data from the positive controls were not used in the statistical analysis. ND—not determined.

**Table 6:** Effects of cell-free supernatant of potential probiotic bacteria isolates on food pathogens.

Isolate	Inhibition Zone (mm)				
	*B. cereus* DSM4384	*B. cereus* F4810/72	*Staphylococcus* *aureus*	*Salmonella enterica* subsp. *enterica*	*E. coli * O157:H7
AD22	11.0 ± 0.02	9.0 ± 0.05 *	7.0 ± 0.01	12.0 ± 0.03	9.0 ± 0.10 *
AD62	14.0 ± 0.14 *	13.5 ± 0.14 *	6.0 ± 0.02 *	10.0 ± 0.14 *	9.5 ± 0.07 *
AD72	13.0 ± 0.14 *	10.5 ± 0.07 *	8.5 ± 0.07	12.0 ± 0.14	11.0 ± 0.14 *
AD73	11.0 ± 0.05	12.0 ± 0.12 *	9.5 ± 0.07 *	13.0 ± 0.10 *	8.0 ± 0.01
AD85	12.0 ± 0.05 *	11.0 ± 0.15 *	8.5 ± 0.07	8.0 ± 0.14 *	7.0 ± 0.12 *
AD118	8.0 ± 0.07 *	7.5 ± 0.07 *	7.0 ± 0.06	9.5 ± 0.07 *	8.0 ± 0.05
Penicillin	21.0 ± 0.05	20.0 ± 0.03	22.0 ± 0.05	ND	ND
Polymyxin B	ND	ND	ND	16.0 ± 0.07	15.0 ± 0.07

* Statistically significant different compared with the other potential probiotic isolates (*p* < 0.05; Tukey’s HSD test with One-Way ANOVA). The data from the positive controls were not used in the statistical analysis. ND—not determined.

**Table 7:** Physical analysis of chèvre cheese.

Characterisation	Chèvre Cheese Made with Starter Culture *	Chèvre Cheese Made with Starter Culture * and Probiotic
Raw goat’s milk	12 kg	12 kg
Whey volume	7.19 L	7.19 L
Weight of cheese	2.185 kg	2.010 kg
Percent yield of cheese (by weight of goat’s milk)	18.20%	16.75%
Moisture content	64.53 ± 0.62% **	61.33 ± 0.68% **
Colour ***	white	white
Texture of cheese ***	firm, homogenous and smooth texture	firm but not completely homogenous texture (lumps found in curd)

* R-704 is a mesophilic starter culture containing *Lactococcus lactic* subsp. *cremoris* and *L. lactic* subsp. *lactis*; ** the data are given as mean ± SD of the results in triplicates; *** evaluated during the manufacturing process.

**Table 8:** The pH and microbiological analysis of chèvre cheese made with starter culture R-704.

Day	pH	Total Viable Count (log CFU/g)	Lactic Acid Bacteria * (log CFU/g)	Yeast and Mould Count ** (log CFU/g)	Detection of Deviated Smell from Day 0 ***
0	4.42	9.84 ± 0.02	10.03 ± 0.02	2.31 ± 0.01	NA
2	4.38	9.81 ± 0.20	9.40 ± 0.03	2.27 ± 0.25	ND
4	4.36	8.82 ± 0.02	8.47 ± 0.03	3.17 ± 0.02	ND
6	4.35	8.38 ± 0.02	7.92 ± 0.05	4.53 ± 0.02	ND
8	4.33	8.37 ± 0.04	7.01 ± 0.03	5.23 ± 0.02	ND
10	4.30	7.99 ± 0.11	6.92 ± 0.01	6.02 ± 0.01	D (alcoholic smell)
12	4.25	8.52 ± 0.05	6.86 ± 0.01	6.35 ± 0.05	D (alcoholic smell)
14	4.28	6.39 ± 0.04	6.61 ± 0.01	6.61 ± 0.01	D (alcoholic smell)

Note: * largely contributed by R-704 starter culture. ** Only yeasts were recovered from DRBC. The data presented in the figure are given as mean ± standard deviation (SD) of triplicate experiments. *** NA—not applicable; ND—not detected; D—detected (evaluated by a sensory panel).

**Table 9:** The pH and microbiological analysis of probiotic chèvre cheese made with starter culture R-704 and probiotic culture *Lactobacillus plantarum* AD73.

Day	pH	Total Viable Count (log CFU/g)	Number of Lactic Acid Bacteria (log CFU/g)			Yeast and Mould Count ** (log CFU/g)	Detection of Deviated Smell from Day 0 ***
			Total Lactic Acid Bacteria	LAB Starters	*L. plantarum* AD73 *		
0	4.36	9.81 ± 0.02	10.49 ± 0.01	10.49 ± 0.01	8.63 ± 0.04	2.16 ± 0.28	NA
2	4.36	9.76 ± 0.04	9.28 ± 0.04	9.22 ± 0.04	8.41 ± 0.08	2.57 ± 0.23	ND
4	4.35	8.65 ± 0.03	9.19 ± 0.05	8.61 ± 0.03	7.83 ± 0.13	3.12 ± 0.04	ND
6	4.35	8.59 ± 0.04	8.33 ± 0.01	8.18 ± 0.01	7.77 ± 0.05	4.00 ± 0.01	ND
8	4.36	8.39 ± 0.08	8.00 ± 0.03	7.81 ± 0.03	7.56 ± 0.05	5.64 ± 0.02	ND
10	4.37	8.15 ± 0.09	7.91 ± 0.02	7.70 ± 0.02	7.49 ± 0.08	5.98 ± 0.01	D (alcoholic smell)
12	4.37	8.33 ± 0.03	7.01 ± 0.03	6.81 ± 0.01	6.58 ± 0.09	6.65 ± 0.02	D (alcoholic smell)
14	4.35	6.76 ± 0.03	6.82 ± 0.01	6.56 ± 0.04	6.46 ± 0.02	6.79 ± 0.03	D (alcoholic smell)

Note: * Probiotic culture. ** Only yeasts were recovered from DRBC. The data presented in the figure are given as mean ± SD of triplicate experiments. *** NA—not applicable; ND—not detected; D—detected (evaluated by a sensory panel).

## Data Availability

This article contains all of the data that was created or evaluated throughout the investigation.
